# A comparison of electrospinning and pressurized gyration: Production of empagliflozin-loaded polylactic acid/polycaprolactone fibrous patches

**DOI:** 10.1098/rsif.2024.0635

**Published:** 2025-03-12

**Authors:** Humeyra Betul Yekeler, Ilke Kabaoglu, Ece Guler, Manuel Pedro F. Graça, Oguzhan Gunduz, Deepak M. Kalaskar, Muhammet Emin Cam

**Affiliations:** ^1^ Department of Pharmacology, Faculty of Pharmacy, Marmara University, Istanbul 34854, Turkey; ^2^ UCL Division of Surgery and Interventional Science, Royal Free Hospital Campus, University College London, London NW3 2PF, UK; ^3^ MecNano Technologies, Cube Incubation, Teknopark Istanbul, Istanbul 34906, Turkey; ^4^ Center for Nanotechnology and Biomaterials Application and Research, Marmara University, Istanbul 34722, Turkey; ^5^ Department of Pharmacology, Faculty of Pharmacy, Istanbul Kent University, Istanbul 34406, Turkey; ^6^ ResearchKent, Istanbul Kent University, Istanbul 34406, Turkey; ^7^ I3N and Physics Department, University of Aveiro, Aveiro 3810-193, Portugal; ^8^ Department of Metallurgy and Material Engineering, Faculty of Technology, Marmara University, Istanbul 34722, Turkey; ^9^ Biomedical Engineering Department, University of Aveiro, Aveiro 3810-193, Portugal

**Keywords:** type 2 diabetes mellitus, empagliflozin, electrospinning, pressurized gyration, transdermal

## Abstract

Novel therapeutic strategies are essential for enhancing efficacy and accelerating the treatment of diabetes mellitus. This investigation focused on incorporating empagliflozin into a composite of polylactic acid and polycaprolactone, resulting in the fabrication of drug-loaded fibrous patches (DFPs) for transdermal application, both by electrospinning (ES) and by pressurized gyration (PG). Scanning electron microscopy results revealed that DFPs generated through the PG method exhibited smaller diameters and a larger surface area than ES. Fourier-transform infrared spectroscopy and X-ray powder diffraction analyses confirmed the successful encapsulation of the drug in both DFPs. DFPs/PG exhibited a controlled release of 98.7 ± 1.3% of the total drug over 14 days, while DFPs/ES released 98.1 ± 2.1% in 12 days, according to *in vitro* drug release studies. This study underscores that the PG method can generate DFPs with extended controlled release. 3-(4,5-dimethylthiazol-2-yl)-2,5 diphenyl tetrazolium bromide test results validate the biocompatibility of DFPs, affirming their lack of adverse effects on human dermal fibroblast cell viability. Consequently, DFPs can be manufactured for transdermal administration using PG, exhibiting similar characteristics to ES but with the added advantage of mass production capability.

## 1. Introduction

In the last few decades, nano-sized fibrous patches (FPs) have been extensively used in drug delivery systems owing to their capability to retain drug molecules in the polymer structure. Numerous technologies have been developed for the production of FPs such as electrospinning (ES), centrifugal spinning, flash spinning, drawing technique, etc. To date, one of the most widespread and preferred methods among these techniques is ES [1,2]. ES is a straightforward, facile and cost-effective process to produce FPs; however, there are some limitations associated with ES [3]. A high-voltage electric field is required to produce electrospun FPs [4]. Also, ES has low production efficiency, high maintenance costs, low FP production per unit of time and low production speed [5].

More recently, alternative methods have been developed to eliminate the shortcomings of commonly used electric field techniques. Pressurized gyration (PG) is a novel, multi-faceted and promising technique that produces FPs by rotating the polymer solution without an electric field. In this study, FPs were fabricated by the PG method. The PG method uses both centrifugal spinning force and gas pressure to generate FPs. A volume of 1 ml of the mixture solution was inserted into a 41 × 60 mm stainless steel vessel with numerous 1.0 mm diameter circular holes (electronic supplementary material, figure S1). FP productions were carried out under a range of conditions (30–60% humidity, 20–40°C) with a rotation speed between 6000 and 12 000 r.p.m. and a working pressure between 0.1 and 0.4 MPa [6]. PG has a large number of finely tuned parameters and greater control over the final product morphology can be achieved. It also has a much larger FP production capacity than ES and its production efficiency is quite high [7,8].

In many of the biomedical applications of FPs, polymers are used as matrices to include therapeutic agents into formulations [9]. Polylactic acid (PLA) is a broadly used polymer in drug delivery systems because of its high biodegradability, biocompatibility, simplicity and environmental friendliness [10,11]. Nevertheless, PLA’s lack of elasticity and brittleness significantly restrict its use in the production of composite FPs [12]. Therefore, blending polymers that have rigid and hydrophobic properties, such as PLA, with two or more polymers at specific ratios is an effective way to produce FPs with superior mechanical and physico-chemical properties [13]. Poly(ε-caprolactone) (PCL) has high ductility and flexibility as well as biodegradability, biocompatibility and low toxicity [14,15]. By these properties, blending PCL with PLA improves the mechanical characteristics of pure PLA by increasing fluidity, impact resistance and flexibility [12]. Through this polymer blending, the rate of release can be altered by changing the percentage of polymers in the mixture, which is also beneficial for the design of controlled drug release formulations [9,16,17]. Furthermore, degradation products of PLA are reported to decrease local pH, enhance the degradation of polyester polymers and stimulate inflammatory responses. From this point of view, blending with PCL has the additional benefit of decreasing the inflammatory response by reducing local acidification to minimization [18,19].

Currently, most active pharmaceutical ingredients are produced as tablets or capsules for oral intake [3]. Empagliflozin (EMPA) is a well-known sodium-glucose cotransporter-2 (SGLT-2) inhibitor currently used orally as a treatment for type 2 diabetes mellitus (T2DM) [20]. Unfortunately, oral administration of EMPA has some shortcomings, such as undergoing first-pass metabolism and reduced intestinal bioavailability, and the insufficient ability to regulate the absorption rate or control drug delivery [21]. To overcome this, polymeric FPs have been used in the transdermal drug delivery systems development, especially in the formulation of poorly water-soluble drugs [22,23]. Through transdermal drug delivery systems, drugs can bypass hepatic first-pass metabolism. As a result, gastrointestinal side effects are reduced while systemic bioavailability is increased. Moreover, the ability to adjust the release rate reduces the frequency of dosing and generally increases patient compliance [24]. Many factors need to be taken into account before designing transdermal delivery systems for drugs, for instance, the skin barrier only permits penetration of molecules of hydrophobic drugs that have a total molecular weight of less than 500 kDa (kilodaltons), and the rate at which drugs cross this barrier is extremely slow [25]. Despite these limitations, EMPA is a suitable agent for the transdermal route due to its low molecular weight and hydrophobic nature.

This work aimed to compare two different FP production techniques, ES and PG. The production qualities and capacities of these techniques were evaluated and their advantages and disadvantages were determined. To this aim, pure PLA/PCL FPs (PFPs) and drug-loaded PLA/PCL FPs (DFPs) were fabricated using ES and PG. The characterization and efficiency of PFPs and DFPs were investigated and compared with Fourier-transform infrared spectroscopy (FTIR), scanning electron microscopy (SEM), tensile test, X-ray powder diffraction (XRD), differential scanning calorimetry (DSC), swelling test, drug encapsulation efficiency (EE), *in vitro* drug release kinetic assay and *in vitro* drug release kinetics. Cell viability was evaluated using the 3-(4,5-dimethylthiazol−2-yl)-2,5 diphenyl tetrazolium bromide (MTT) cell proliferation assay.

## 2. Material and methods

Material and methods for preparation and characterization of solutions, preparation and characterization of FPs, SEM, FTIR, XRD, DSC, tensile test, drug EE, *in vitro* release kinetic assay, swelling test, *in vitro* cytotoxicity test, and statistical analysis are given in electronic supplementary material.

## 3. Results

### 3.1. Physical characteristics of solutions

**Figure 1.**
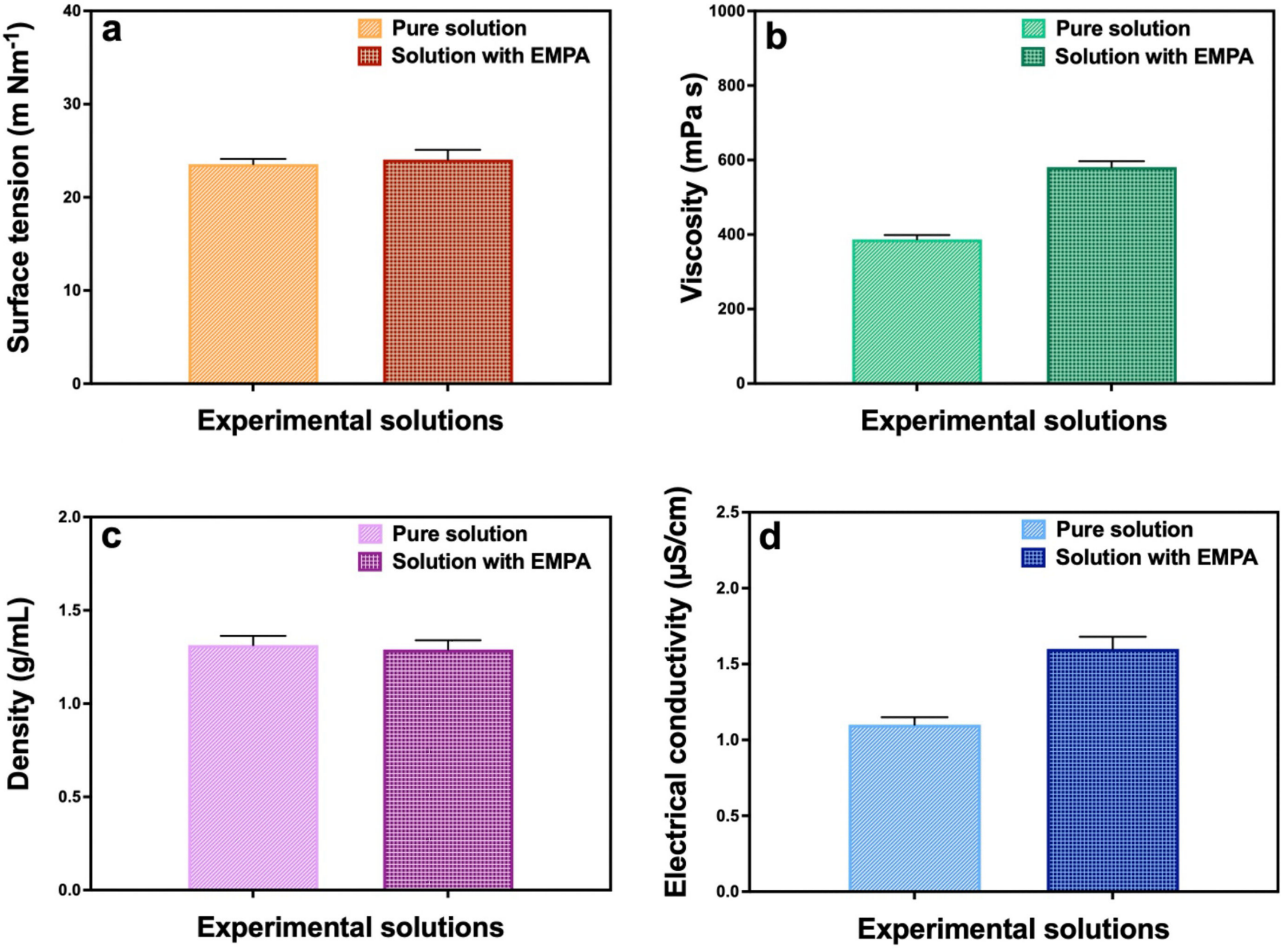
Physical properties for each solution utilized in the experiment: (*a*) surface tension, (*b*) viscosity, (*c*) density, and (*d*) electrical conductivity (*n* = 3).

The physical characteristics of solutions (electrical conductivity, surface tension, densities and viscosities) were measured (figure 1). According to the results obtained, a slight increase in surface tension was noted by the addition of EMPA to the pure PLA/PCL (8% PLA, w/v; 15% PCL w/v) solution. While the surface tension increased from 23.6 to 24.1 mN m^−1^, the density decreased from 1.31 to 1.29 g ml^−1^. Besides, the viscosity increased from 386.9 to 581.0 mPa s, and the electrical conductivity increased from 1.1 to 1.6 µS cm^−1^. After the incorporation of EMPA into the pure solution, an increase in the diameter of the FPs was observed according to SEM images. Also, the addition of EMPA led to higher values for surface tension, viscosity and electrical conductivity in the pure solution. These findings were consistent with the previous literature [26].

### 3.2. Morphological characterization of fibrous patches

**Figure 2.**
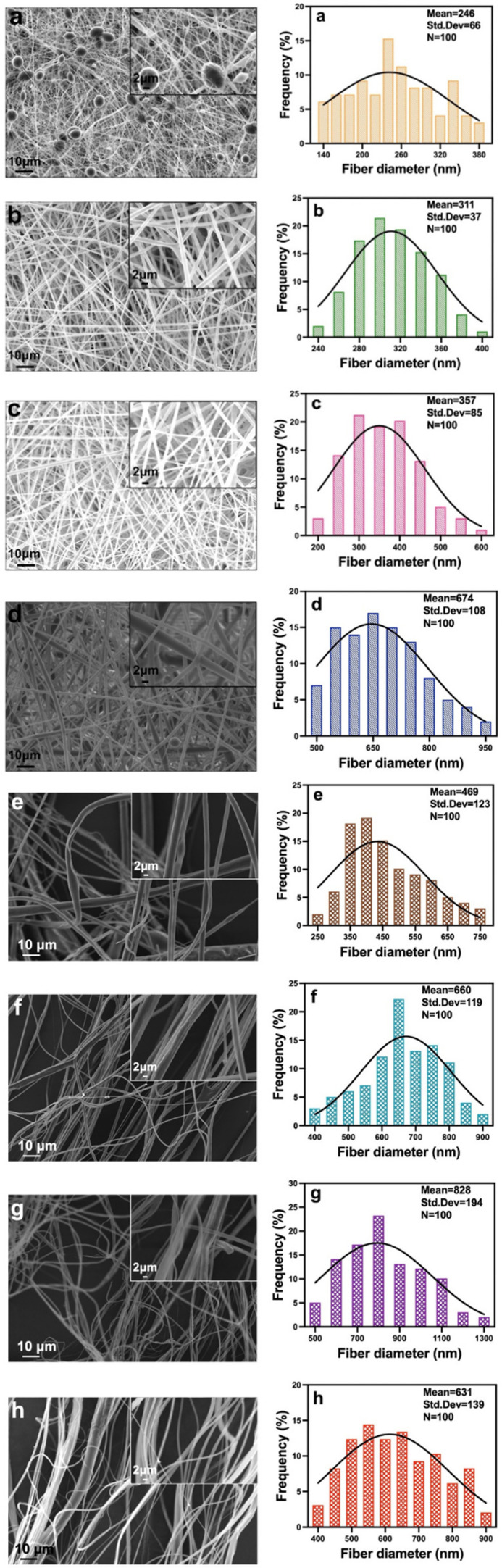
SEM images and FPs diameter distributions of the PFPs and DFPs produced by ES (*a–d*) and PG (*e–h*). (*a*) pure PLA/PCL (1 : 1, v/v) FP, (*b*) pure PLA/PCL (3 : 1, v/v) FP, (*c*) pure PLA/PCL (5 : 1, v/v) FP, (*d*) EMPA-loaded PLA/PCL (3 : 1, v/v) FP, (*e*) pure PLA/PCL (1 : 1, v/v) FP, (*f*) pure PLA/PCL (3 : 1, v/v) FP, (*g*) pure PLA/PCL (5 : 1, v/v) FP, and (*h*) EMPA-loaded PLA/PCL (3 : 1, v/v) FP. In all diameter distributions, *n* = 100.

To observe changes in the morphology of PFPs and DFPs, SEM images of all FPs were examined (figure 2). Comparisons of PFPs formed using different ratios of PLA and PCL were made to obtain the best resulting FPs. When producing FPs with ES, the voltage values ranged from 14 to 28 kV, while the flow rate was between 0.05 and 3.2 ml h^−1^. In addition, the distance was 150 mm from the tip of the needle to the metal plate. While an increase was observed in the applied voltage value as the PLA ratio increased, when EMPA was added to the 3 : 1 PLA/PCL solution, there was a noticeable decrease in both voltages (26.8–14.1 kV) and flow rate (2.2–0.05 ml h^−1^). While producing FPs with PG, the temperature was kept between 26°C and 28°C and the relative humidity was 35–50%. As the PLA concentration increased, an increase in humidity (37–50%) and a decrease in temperature (27.5–26°C) were observed.

The diameters of PFPs produced by ES were 246 ± 66 nm in a 1 : 1 ratio, 311 ± 37 nm in a 3 : 1 ratio and 357 ± 85 nm in a 5 : 1 ratio. The diameters of PFPs produced by PG were 469 ± 123 nm in a 1 : 1 ratio, 660 ± 119 nm in a 3 : 1 ratio and 828 ± 194 nm in a 5 : 1 ratio. As a result of the evaluations, the FPs with the most ideal morphology in both methods were determined as the FPs in which PLA/PCL was used at a ratio of 3 : 1. Since the structure of the FPs produced using this ratio is homogeneous and bead-free formation was observed, this ratio was used for the continuation of the experiment. Then, after adding EMPA to the 3 : 1 pure solution, there was a noticeable increase in the diameter (from 311 ± 37 to 674 ± 108 nm) of the ES-produced FPs. However, a decrease (from 660 ± 119 to 631 ± 139 nm) was observed in the FPs produced with PG. When we compared the diameters of DFPs, it was observed that the diameters of PG-produced DFPs were smaller. After the patches were produced with the two techniques, morphological characterizations were performed and it was observed that increasing the PLA ratio in the solution caused a rise in the diameter of FPs.

### 3.3. Determination of molecular structure

**Figure 3.**
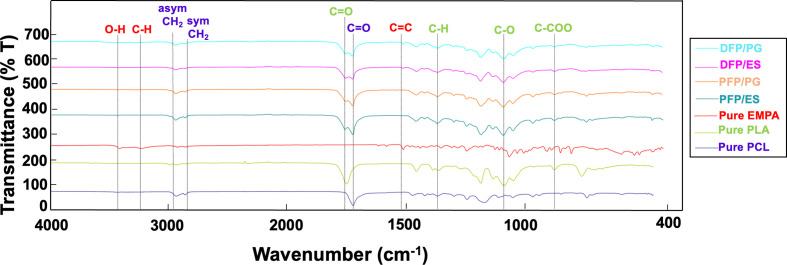
FTIR spectra of pure PCL, PLA, EMPA, PFPs and DFPs produced by ES and PG.

Characterization and molecular structure of pure PLA, PCL, EMPA, PFPs, and DFPs were carried out by FTIR tests. The results are presented in figure 3.

Characteristic absorption bands of pure PLA observed were as follows: C=O stretching band, which can be identified as a backbone ester group of PLA, bending at 1747 cm^−1^, vibration for CH_3_ asymmetric scissoring at 1452 cm^−1^, ester C–O asymmetric stretching at 1180 cm^−1^, C–COO stretching peak at 869 cm^−1^, bending vibrations for CH_3_ and C–H at 1382 and 1359 cm^−1^, stretching vibrations for CH_3_ and C–H at 2996 and 2945 cm^−1^, respectively [27,28].

FTIR spectra of pure PCL show a peak at 1720 cm^−1^ related to C=O stretching. The characteristic bands are the C–H group bending at 1470 and 1416 cm^−1^, and C–O stretching in the crystalline phase at 1293 cm^−1^. Besides, CH_2_ symmetric and asymmetric stretching at 2865 and 2940 cm^−1^, C–O–C symmetric and asymmetric stretching at 1164 and 1238 cm^−1^, O–H bending at 1396 and 1364 cm^−1^ were observed [29].

Characteristic infrared bands for pure EMPA, a band in the region with a peak of 1508 cm^−1^ was observed, corresponding to the aromatic C=C group; 3419 cm^−1^ is from the O–H stretching; 3237 cm^−1^ is from the aromatic C–H stretching. Other peaks were present: C–O–C stretching band at 1240 cm^−1^, =C–H stretching band at 2952 cm^−1^, C–Cl stretching band at 796 cm^−1^, C–H stretching band at 2931 cm^−1^, CH2 stretching band at 2867 cm^−1^, C=C stretching band at 1614 cm^−1^, C–H bending at 1436 cm^−1^ and C=O stretching band at 1063 cm^−1^ [30].

The spectra of DFPs produced by both methods show that the peaks at 1720 and 2940 cm^−1^ belong to PCL and the peaks at 1747, 1180, 1128 and 869 cm^−1^ belong to PLA. In comparison with the absorption spectra of pure PLA and PCL, the weaker peak for the aromatic C=C group stretching at 1508 cm^−1^ in both DFPs proves the presence of EMPA. These findings indicate that effective drug encapsulation and formulation were accomplished.

### 3.4. Determination of crystallinity

**Figure 4.**
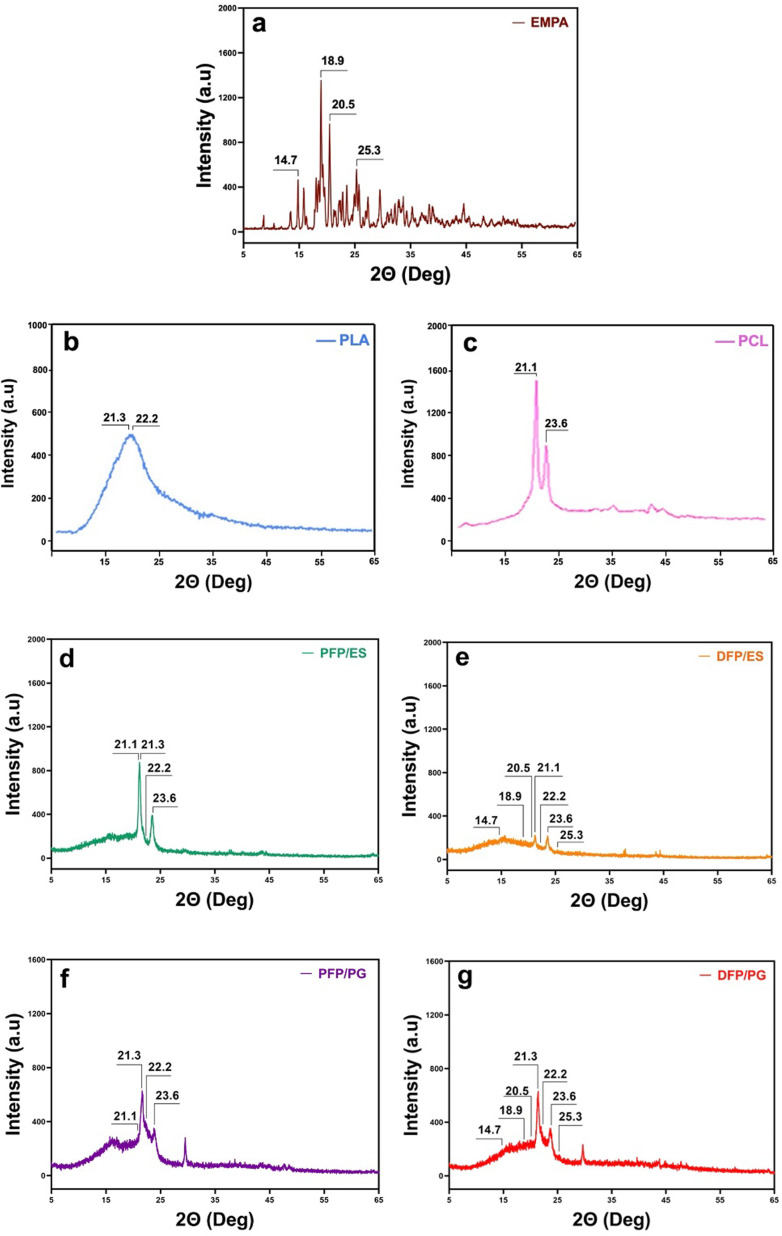
XRD patterns of (*a*) pure EMPA, (*b*) pure PLA, (*c*) pure PCL, (*d*) PFPs produced ES, (*e*) DFPs produced by ES, (*f*) PFPs produced by PG, and (*g*) DFPs produced by PG.

To understand whether EMPA was successfully loaded into PFPs and to examine the structures of PLA/PCL, XRD analysis was performed. The XRD results of PFPs produced with ES and PG are shown in figure 4. Pure EMPA exhibited characteristic peaks at 14.7°, 18.9°, 20.5° and 25.3° 2θ degrees; these results showed similar 2θ degrees values as the crystalline form of EMPA. The sharp peaks seen for PLA were 21.3° and 22.2° 2θ, while the peaks seen for PCL were 21.1° and 23.6° 2θ. Similar peaks were observed in the XRD results of DFPs produced by ES and PG. We observed the characteristic peaks of EMPA in the DFPs were weakened when compared with pure EMPA. The weakening of the characteristic peaks of EMPA in the XRD diffractogram of DFPs indicates that the crystal structure of EMPA is weakened when added to FPs. Consequently, the characteristic peaks of PLA, PCL and EMPA were seen in DFPs. According to these results, it was confirmed that EMPA was successfully encapsulated in DFPs.

### 3.5. Thermal analysis

**Figure 5.**
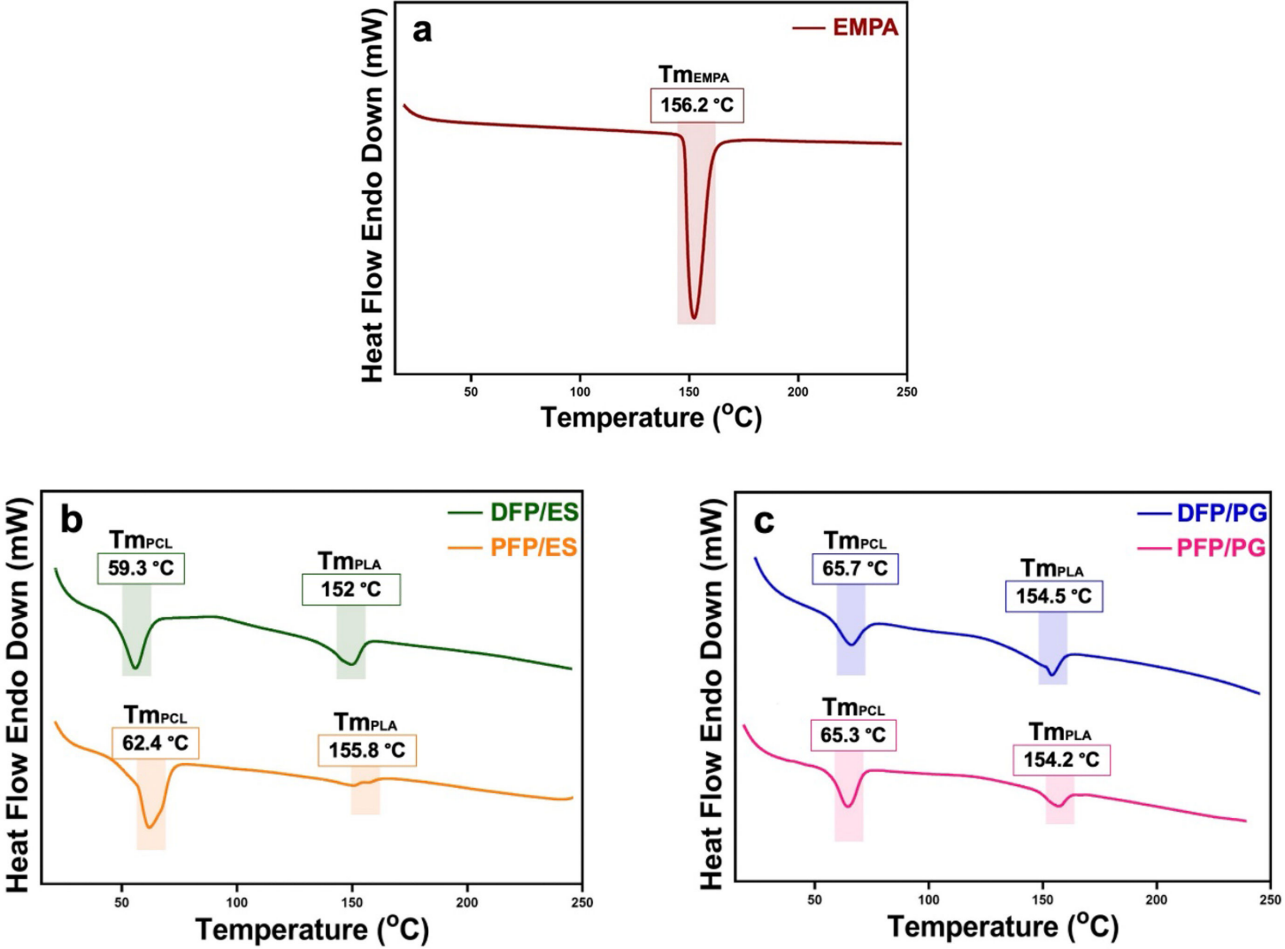
DSC curves of (*a*) pure EMPA, (*b*) PFPs and DFPs produced by ES and (*c*) PFPs and DFPs produced by PG.

The purpose of DSC tests is to understand how the materials used in FP production affect properties including glass transition (Tg) and melting temperature (Tm). Additionally, the differences between the production methods used were also examined. Figure 5 shows DSC curves of PFPs and DFPs within the temperature range 0–300°C. While the observed Tg and Tm values for PLA were 61°C and 154°C, respectively, these values were observed as −60°C and 60°C for PCL. The Tg value of PCL (−60°C) was not recorded in the temperature range we studied [31]. In the DSC curve of PFP with ES and PG, peaks were seen at 62.4°C and 65.3°C. In addition, the Tm value of PLA for ES and PG was found at 155.8°C and 154.2°C, respectively. Whereas the Tg value was not observed for EMPA, the apparent endothermic peak for the Tm value was 156.2°C. Tm (59.3°C) and Tg (152 °C) values decreased when EMPA was added to PFPs, which is produced by ES. On the other hand, Tg and Tm values remained almost the same on PFPs produced with PG and ES. The results of the samples produced by ES and PG did not show any significant difference.

### 3.6. Tensile testing of fibrous patches

**Figure 6.**
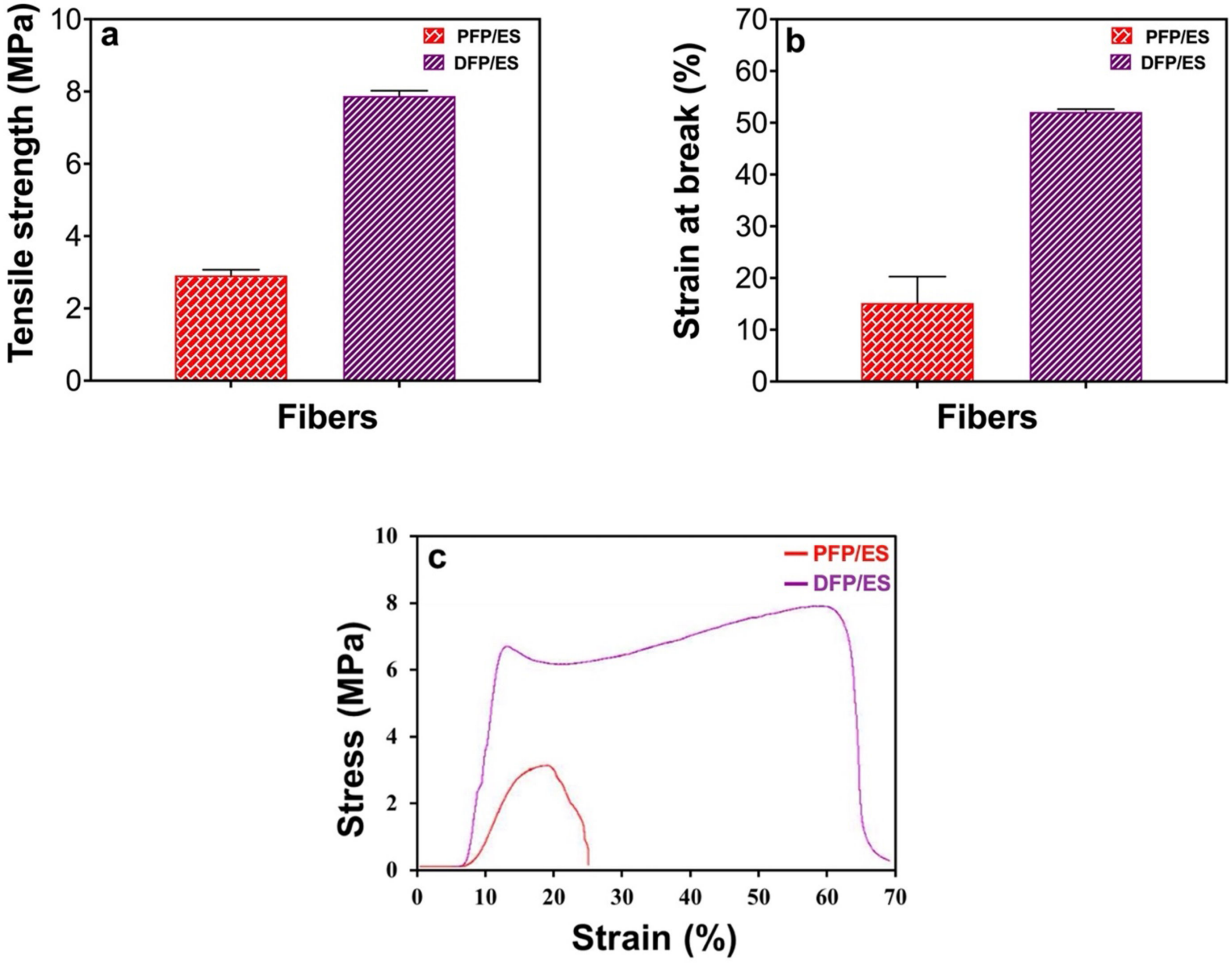
Physical parameters of PFPs and DFPs produced by ES. (*a*) Tensile strength, (*b*) strain at break, and (*c*) stress–strain curve (*n* = 3).

Various measurements were made to understand how the loading of EMPA on PFPs affected the physical properties of the DFPs. Among these measurements, properties such as tensile strength and strain at break were examined and then a stress–strain curve was created. According to the results, after EMPA loading, the tensile strength increased from 2913.0 ± 156.3 kPa to 7878.1 ± 145.1 kPa, the strain at break increased from 15.2 ± 5.1% to 52.1 ± 0.6%. As a result, the mechanical properties of FPs increased by adding EMPA. In our study, it was found that the tensile strength increased with the increase in FP diameter. As can be seen from the stress–strain curves, the tensile properties increased with the addition of EMPA (figure 6) [32].

### 3.7. *In vitro* drug release test

**Figure 7.**
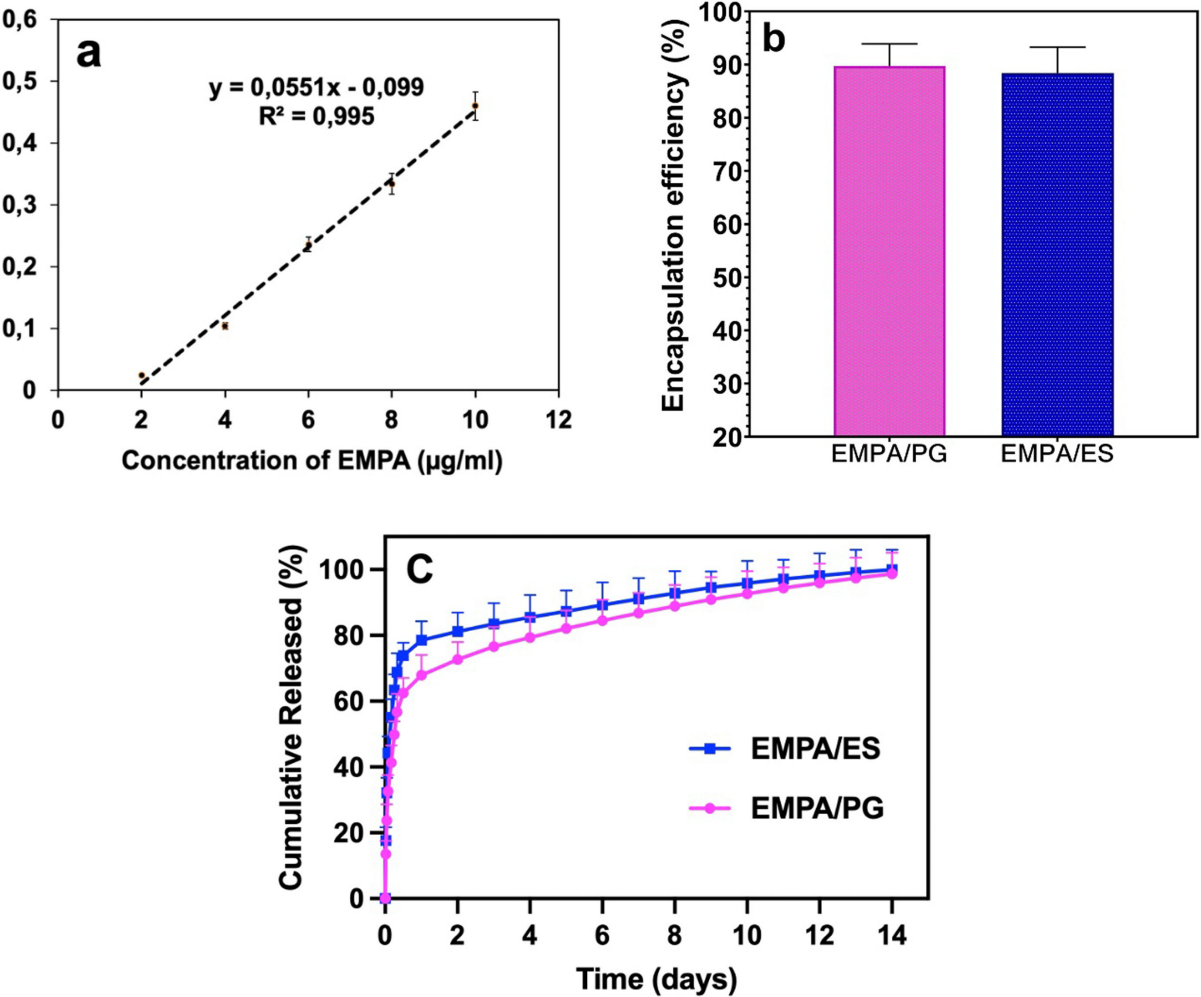
*In vitro* drug release profile of DFPs produced by ES and PG (*a*) EMPA calibration curve, (*b*) encapsulation efficiency of DFPs, and (*c*) EMPA release profiles within 14 days. Each measurement was performed with three repetitions and the errors were less than 5% (*n* = 3).

Firstly, five different EMPA concentrations between 2 and 10 μg ml^−1^ were generated and their UV spectra were analysed. The EMPA absorption values (*R*^2^ = 0.995) obtained for the quantitative measurement of drug release were used to generate the linear standard calibration curve (figure 7a). The released EMPA was detected at 223 nm by UV [33,34]. In addition, the EE of DFPs produced by PG was calculated at 89.7 ± 4.1%, and the EE of DFPs produced by ES was calculated at 88.4 ± 3.9% (figure 7b). Then, *in vitro* release experiments were performed to measure the release behaviour of encapsulated EMPA within 14 days for DFP/PG and DFP/ES. The experiments were conducted under the sink conditions [17]. The releasing profile of DFP/PG and DFP/ES was examined in phosphate-buffered saline (PBS) (pH 7.4, 37°C) to imitate the physiological conditions of the living organisms. Figure 7c shows the drug release profile of DFP/PG and DFP/ES. While DFP/PG and 56.7 ± 1.8% of EMPA were dissolved in the first 8 h, DFP/ES and 55.1 ± 2.5% of EMPA were released into the dissolution medium in the first 4 h. In addition, while 98.7 ± 1.3% of the drug in FPs produced by PG was successfully released in 14 days in a controlled manner, this rate was 98.1 ± 2.1% of the drug in ES in 12 days.

### 3.8. *In vitro* drug release kinetics

**Figure 8.**
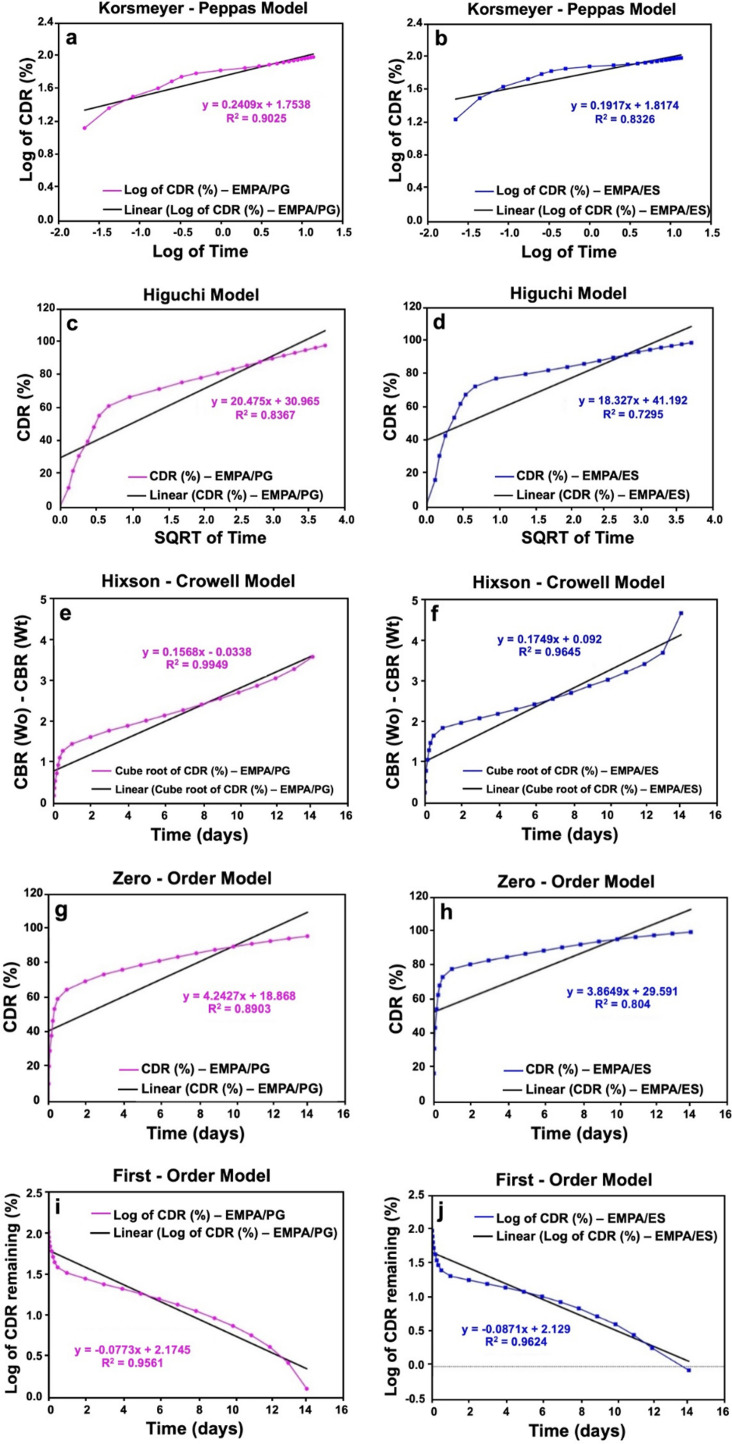
The release kinetic models of DFPs produced by ES and PG: (*a*) Korsmeyer–Peppas model of DFPs produced by PG (EMPA/PG), (*b*) Korsmeyer–Peppas model of EMPA/ES, (*c*) Higuchi model of EMPA/PG, (*d*) Higuchi model of EMPA/ES, (*e*) Hixson–Crowell model of EMPA/PG, (*f*) Hixson–Crowell model of EMPA/ES, (*g*) zero-order model of EMPA/PG, (*h*) zero-order model of EMPA/ES, (*i*) first-order model of EMPA/PG, and (*j*) first-order model of EMPA/ES.

The release kinetics of EMPA from FPs produced by PG and ES were analysed by five main release kinetic models: Korsmeyer–Peppas, Higuchi, Hixson–Crowell, zero order and first order (figure 8). According to the results, the highest *R*^2^ value appeared in the Hixson–Crowell model for both methods. While the *R*^2^ value was 0.9949 for PG (figure 8i), this value was 0.9645 for ES (figure 8j). Moreover, values of *n* corresponding to different transport mechanisms were utilized to characterize the Korsmeyer–Peppas release model. If *n* is less than or equal to 0.45, the transport mechanism is called the Fickian diffusion mechanism, higher than 0.45 and less than 0.89 mechanism is called Fickian transport, equal to 0.89 mechanism is called case II (relaxational) transport, and greater than 0.89 mechanism is called Super case II transport [35]. According to the results, *n* values were below 0.45 for both methods. Since it is observed as 0.1917 for ES and 0.2409 for PG, it was shown that EMPA was released from DFPs produced by PG and ES via the Fickian diffusion mechanism (table 1).

**Table 1.** Data from drug release kinetic models for DFPs.

	Korsmeyer–Peppas		Higuchi		Hixson–Crowell		zero-order		first-order	
**sample**	***R^2^***	***n***	***R^2^***	***K_h_***	***R^2^***	***K_hc_***	***R^2^***	***K_0_***	***R^2^***	***K_1_***
**DFP/PG**	0.9025	0.2409	0.8367	20.475	0.9949	0.1568	0.8903	4.2427	0.9561	−0.0773
**DFP/ES**	0.8326	0.1917	0.7295	18.327	0.9645	0.1749	0.804	3.8649	0.9624	−0.0871

### 3.9. Swelling behaviours of fibrous patches

**Figure 9.**
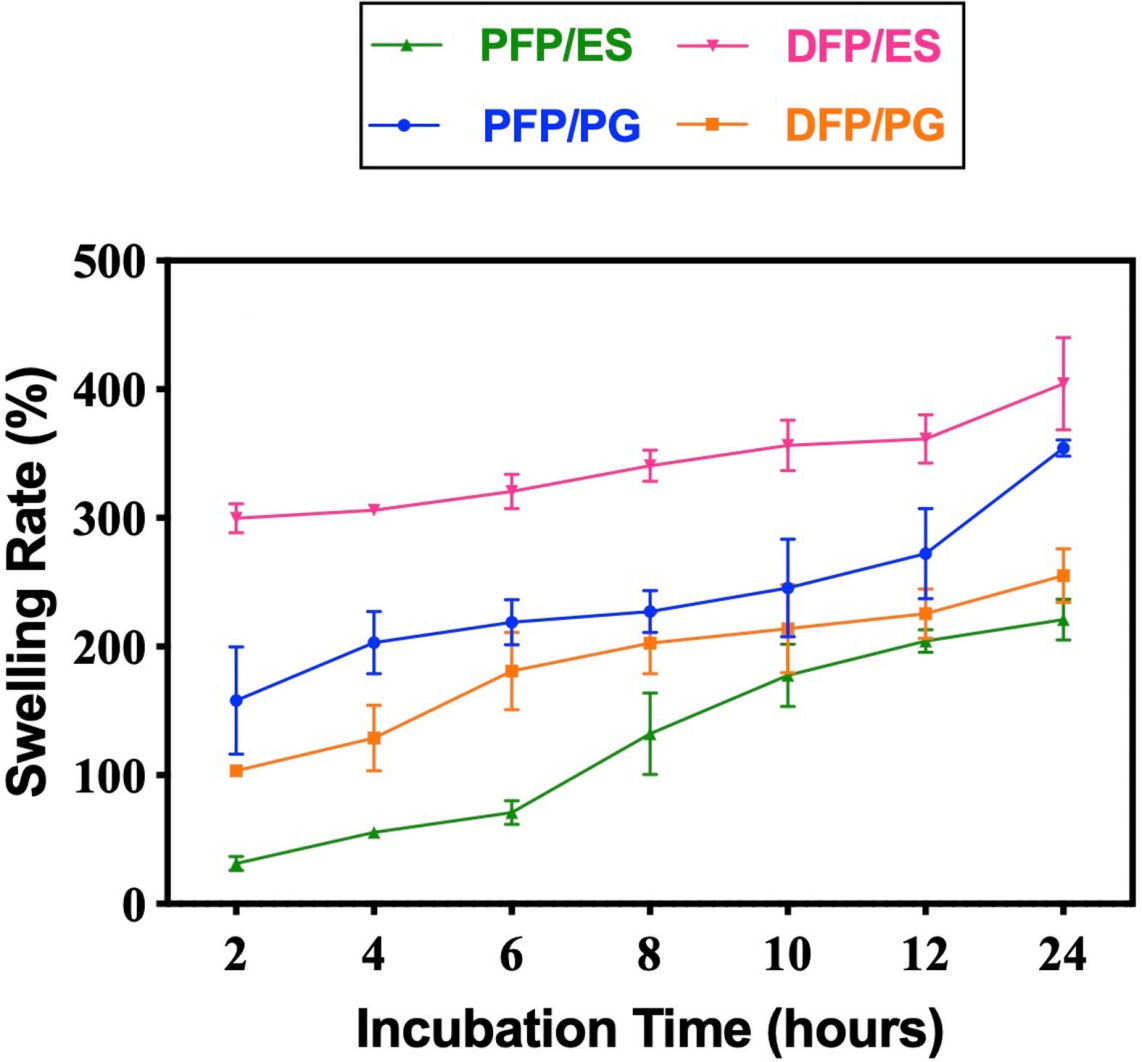
Swelling kinetics of PFPs and DFPs produced by ES and PG (*n* = 3).

Swelling behaviours are important parameters for the characterization of FPs and their applications in biomedical treatments [36]. In addition, the swelling capacity of polymers plays a regulatory role in drug release. Since PLA and PCL polymers are hydrophobic, the amount of water uptake is limited at early time points [37]. The swelling percentages of PFPs and DFPs produced by ES and PG are shown in figure 9. When the swelling ratios were measured, it was observed that the DFPs which were produced by ES had the highest percentage (404%) and it was observed that it was approximately 5 times heavier than the initial weight at the end of 24 h. On the other hand, the lowest percentage was observed in PFPs produced with ES. In addition, it was concluded that PFPs and DFPs produced by PG had 354 and 255% swelling percentages, respectively. According to the result, FP diameters increased as the swelling ratio increased in all four groups. As mentioned in the literature, the increase in FP diameter as the swelling percentage increases is supported by the results of our experiment [38].

### 3.10. Evaluation of cell viability

**Figure 10.**
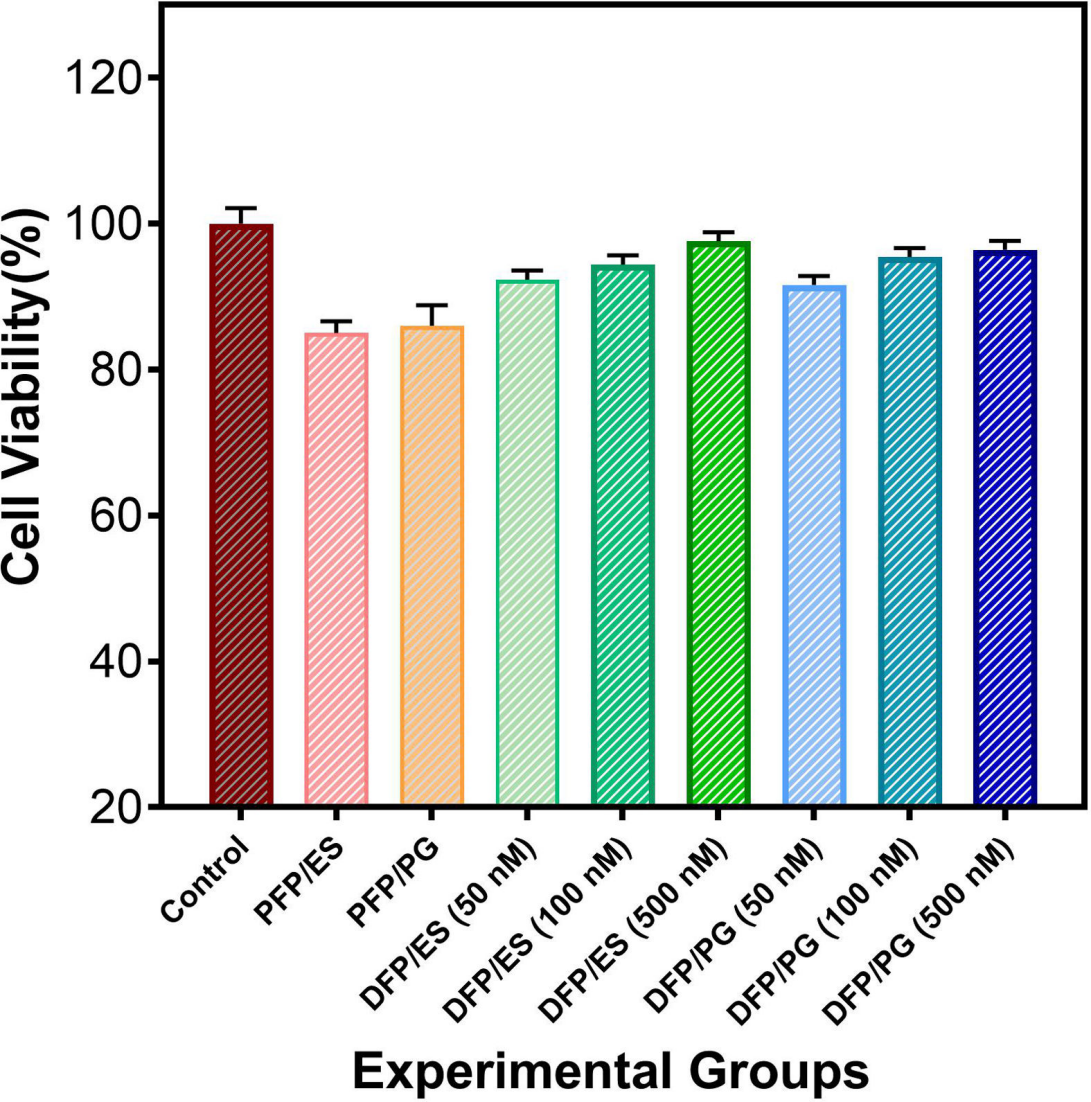
Viability of human dermal fibroblast cells in all samples after 48 h. The mean and standard error of the mean are used to present the data (*n* = 3).

The MTT analysis was used to examine the cytotoxicity profile of all samples in the human dermal fibroblast cell line. The cells treated with growth medium were used as the control group. The DFPs produced by ES and PG were treated at three different concentrations of 50, 100 and 500 nM. As indicated in figure 10, the highest cell viability for DFP/ES and DFP/PG was at 500 nM concentration with 97.6 and 96.4%, respectively. According to the results, we found that cell proliferation *in vitro* increased with increasing drug concentration but there was not any significant difference between groups. Overall, all samples exhibited cell viability of over 85.0% with no significant cytotoxic effect. This suggests that both nanofibre production techniques do not have any negative effect on cell viability.

## 4. Discussion

In this study, DFPs were designed transdermally to offer an effective treatment option for T2DM. Polymer selections were made accordingly and FPs were successfully produced by two different production methods, ES and PG. The characterization of the products and their *in vitro* effects were examined. Moreover, two different FP production methods were compared.

The surface tension of polymer solutions plays a critical part in determining the occurrence of FPs in both techniques. While ES and PG require surface tension to overcome the formation of FPs, increased surface tension may prevent the formation of FPs or cause them to be beaded densely [39]. The surface tension of both solutions was at an acceptable interval for the production of FPs without beading. No substantial difference in the density of PLA/PCL solution was detected with the addition of EMPA. The electrical conductivity increased when EMPA was loaded with the PLA/PCL solution. In ES, high electrical conductivity is desired to produce small-diameter and bead-free FPs [40]. Increased polymer solution concentration and drug content lead to increased viscosity. The viscosity of the solutions affects the morphological properties of FPs in ES and PG. In the ES process, if the concentration and viscosity are higher than desired, the molecular chains of the polymer solution tangle up tightly and therefore are unable to stretch effectively during production. This results in the interruption of the formation of stable and uniform FPs [41]. Similarly in the PG method, the viscosity value has a significant impact on FP diameter, bead size and jet stabilization [42]. In our study, the viscosity increased when we added EMPA to the pure solution as expected; however, both values were in a suitable range to produce FPs successfully.

For the optimization of FP production, pure PLA/PCL composite solutions with three different ratios (1 : 1, 3 : 1, 5 : 1) were prepared, then FPs were produced using ES and PG methods and their morphological properties were compared by SEM. Homogeneous and bead-free FPs with ideal morphology were obtained with PLA/PCL (3 : 1) ratio and chosen as the optimized ratio. Subsequently, DFPs were successfully fabricated by both methods. Although the FP diameter increased with the addition of EMPA to the ES-produced FPs, the morphology of the DFPs was better and they had smooth surface area. On the other hand, as requested, the FP diameters decreased when EMPA was added to the FPs produced by PG. As a result, when comparing the DFPs produced by both methods, it was observed that the PG-produced DFPs had the desired morphological characteristics with lower diameter and higher surface area. Consistent with our previous study [43], we confirmed that the PG method has a much faster and higher production capacity than the ES method.

Based on the tensile test findings, the mechanical properties of FPs increased with the addition of EMPA. This increase may be due to the reaction between the substances in EMPA and the molecules in the structure of the polymers used in FP production [44]. Moreover, it was also found that the tensile strength increased as the FP diameter increased. Based on the earlier studies, the tensile strength is directly related to the FP diameter and the increase in tensile strength may be associated with the increase in FP diameter [45,46].

One of the major concerns of transdermal drug delivery systems is the drug’s degree of crystallinity [47]. Drugs are generally preferred in amorphous form in transdermal patches. This is mainly because the state of amorphous matter dissolves more quickly, becomes more chemically reactive, and absorbs more water vapour compared with the crystalline state. When we examined the XRD diffractograms of our study, we observed that the crystalline structure weakened after the loading of EMPA into the FPs. By reducing the crystallinity, we may be able to increase the dissolution rate, efficiency, and thus bioavailability of EMPA [48].

The results of FTIR and XRD showed that the characteristic peaks of EMPA, PLA and PCL were seen in the DFPs produced by both methods and proved a successful formulation and encapsulation. The results of FTIR and XRD showed that DFPs produced by both methods exhibited characteristic peaks of EMPA, PLA and PCL, proving a successful formulation and encapsulation. The Tm value of EMPA was not observed in the DSC curve of DFPs. This may be proof of the homogeneous distribution of EMPA in DFPs and the intermolecular interaction between EMPA and PLA/PCL [49]. All observed Tm and Tg values were in line with earlier studies [50–52]. When the DSC results of the samples produced using ES and PG were compared, no significant difference was seen. In addition, DSC results prove that the FPs we produced are safe to use for transdermal application since they do not melt at body temperature.

*In vitro* drug release studies showed that DFPs produced by both methods encapsulated EMPA in a high and nearly similar percentage. DFPs produced with ES showed burst release in the first 4 h, while PG-produced DFPs showed during the first 8 h. Subsequently, PG-produced DFPs presented controlled release behaviour for 14 days, whereas ES-produced DFPs showed it for 12 days. This extended controlled release may be due to the hydrophobic nature of PLA and PCL, slowing down the drug diffusion through the FP matrix [53]. Consequently, we proved that the PG method is capable of producing DFPs with a longer duration of controlled release. Furthermore, it was seen that ES-produced DFPs exhibited greater swelling behaviour compared with PG-produced DFPs. Considering the relationship between the *in vitro* drug release test and the swelling test, we also observed that the ES-produced DFPs release relatively higher amounts of the drug compared with the PG-produced DFPs in the first 24 h, consistent with the swelling assay. According to these results, release and swelling behaviours were found to be directly proportional to each other [54].

The MTT results are supported by the results of many studies showing that increasing doses of EMPA do not have any toxicity on cells [55,56]. The obtained results of this study are promising in terms of shedding light on the necessity of *in vivo* studies and further studies for the transdermal application of DFPs in the treatment of T2DM.

## 5. Conclusions

In order to offer a viable transdermal therapeutic solution for the treatment of T2DM, we generated DFPs through two distinct production methods, namely ES and PG, and conducted a comparative analysis. SEM results revealed that DFPs produced via PG exhibited the desired morphological characteristics, featuring lower diameters and increased surface area. Successful encapsulation of the drug, as indicated by the characteristic peaks of EMPA and polymers, was affirmed through FTIR and XRD analysis in DFPs produced by both methods. DSC results established the safety of the FPs for transdermal administration, as they did not exhibit melting at body temperature. *In vitro* studies on drug release demonstrated that DFPs generated through both methods effectively encapsulated EMPA at high and nearly equivalent rates. Notably, DFPs/PG displayed controlled release behaviour for 14 days, while DFPs/ES maintained it for 12 days. This substantiates that the PG method can produce DFPs with an extended duration of controlled release. Furthermore, it was observed that DFPs/ES exhibited greater swelling behaviour compared with DFPs/PG, and the *in vitro* drug release assay correlated with the swelling assay, with DFPs/ES releasing relatively higher amounts of the drug in the initial 24 h. The results of the *in vitro* cytotoxicity test demonstrated that cells exposed to increasing concentrations of EMPA did not exhibit adverse effects. To conclude, DFPs can be efficiently produced for transdermal administration using the PG method, offering similar features to ES but with the advantages of being capable of mass production, not requiring the use of electric field and having a safer working environment.

## Data Availability

The datasets supporting this article have been uploaded as part of the electronic supplementary material [57].

## Dedication

The authors dedicate this article to the memory of Turkish citizens who lost their lives in the earthquakes in Pazarcık and Elbistan-Kahramanmaraş and Hatay, Türkiye on 6 February 2023.
